# Supplementary material for: Copper-Promoted Cross-Coupling Reactions for the Synthesis of Aryl(difluoromethyl)phosphonates Using Trimethylsilyl(difluoromethyl)phosphonate
Source: Molecules. 2018 Dec 11;23(12):3292. doi: 10.3390/molecules23123292 (PMC6321065; doi:10.3390/molecules23123292)

Supporting Information

for

Copper-Promoted Cross-Coupling Reactions for the Synthesis of Aryl(difluoromethyl)phosphonates Using Trimethylsilyl(difluoromethyl)phosphonate

Kazuki Komoda,[1] Rei Iwamoto,[2] Masakazu Kasumi,[1] and Hideki Amii[1]*

[1] Division of Molecular Science, Graduate School of Science and Technology, 1-5-1, Tenjin-cho, Kiryu, Gunma 376-8515, Japan
[2] Department of Chemistry, Graduate School of Science, Kobe University, Nada-ku, Kobe 657-8501, Japan

^{1}H NMR Spectrum of diethyl (4-cyanophenyl)difluoromethylphosphonate (**3a)**

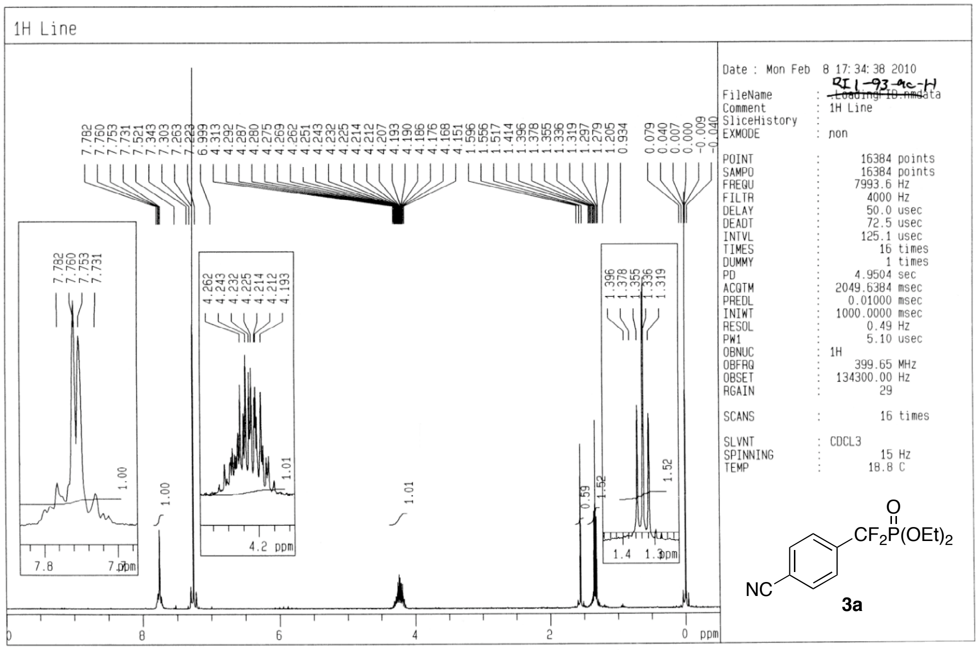

^{19}F NMR Spectrum of diethyl (4-cyanophenyl)difluoromethylphosphonate (**3a**)

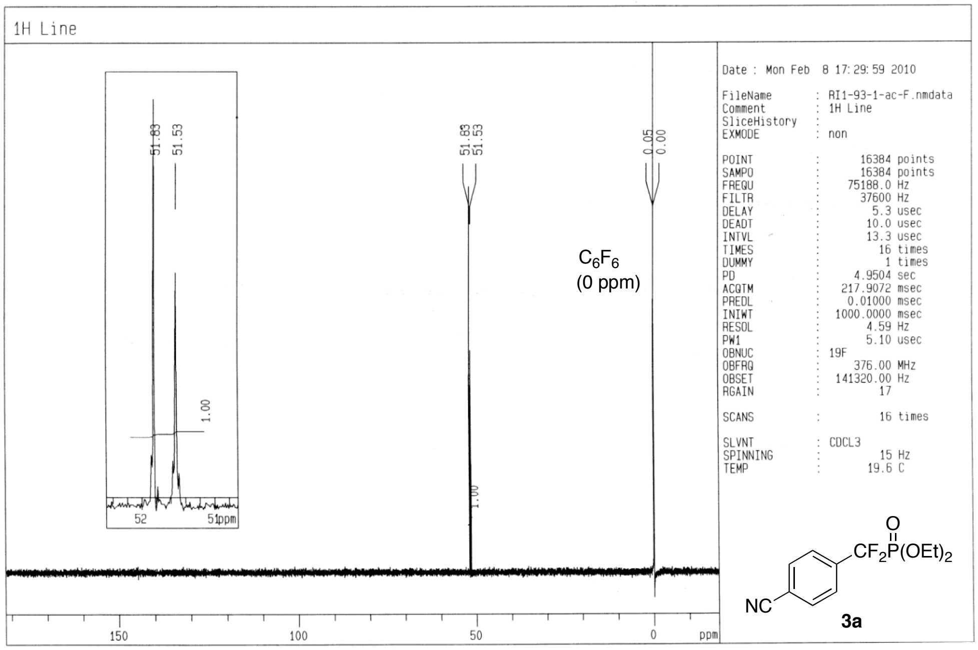

^1H NMR Spectrum of ethyl 4-[(diethoxyphosphoryl[difluoromethyl)benzoate (**3b**)

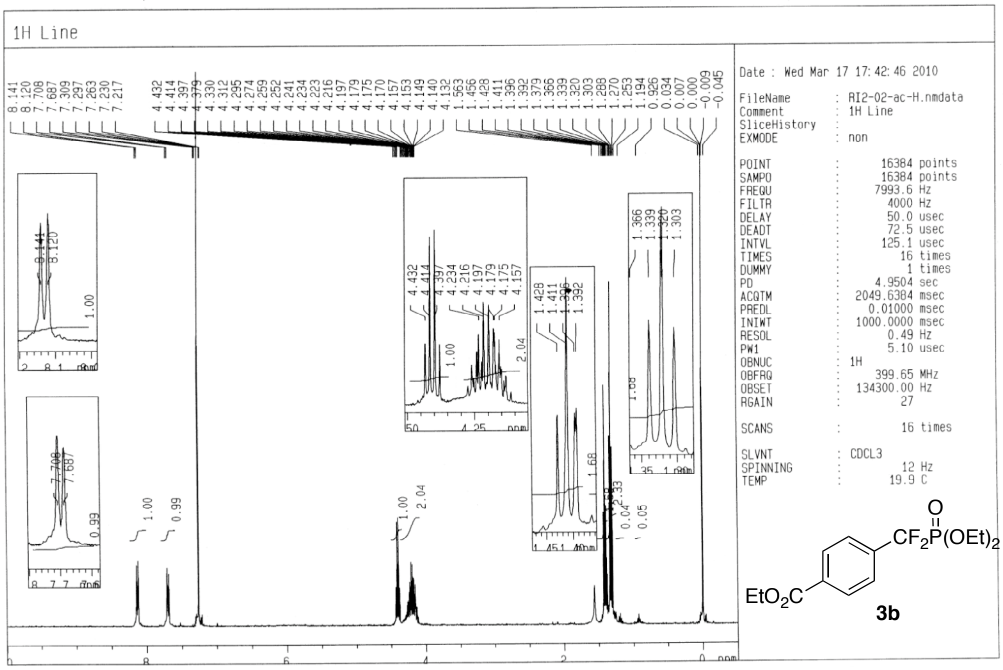

^{19}F NMR Spectrum of ethyl 4-[(diethoxyphosphoryl)difluoromethyl)benzoate (**3b**)

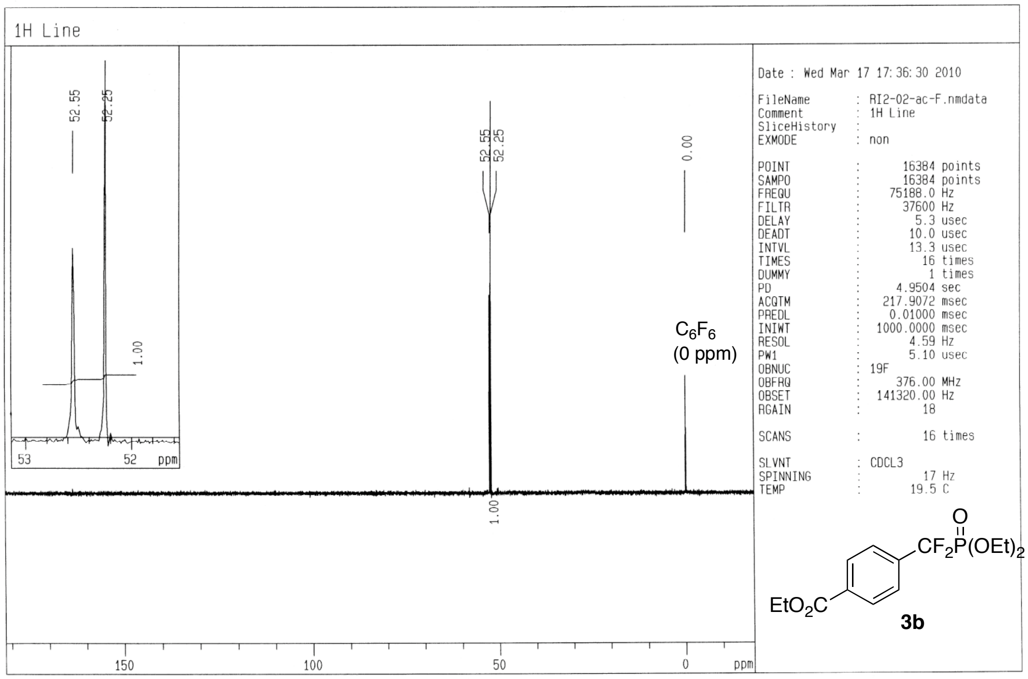

^{1}H NMR Spectrum of diethyl difluoro(phenyl)methylphosphonate (**3c**)

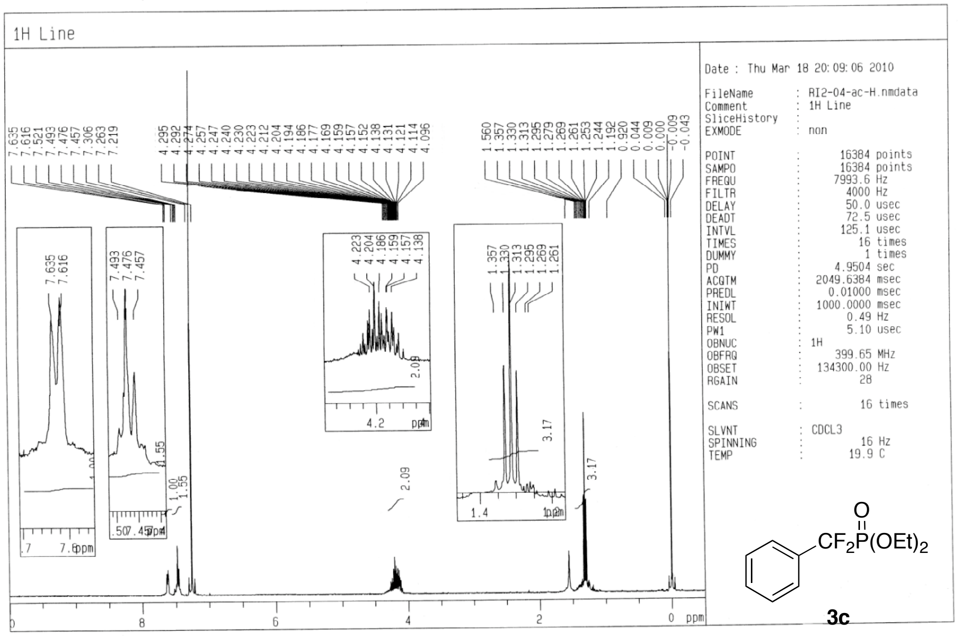

^{19}F NMR Spectrum of diethyl difluoro(phenyl)methylphosphonate (**3c**)

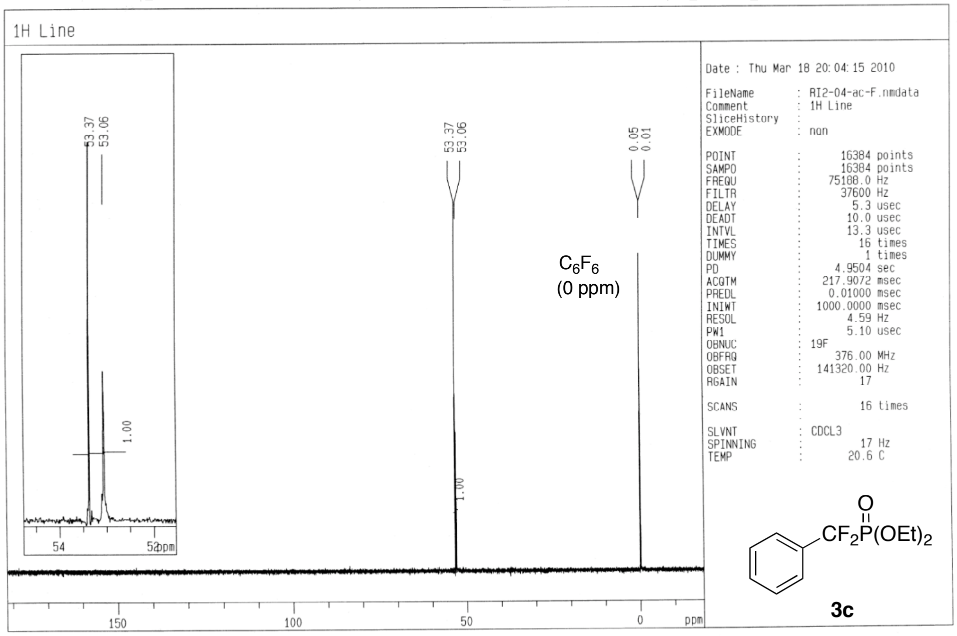

^{1}H NMR Spectrum of diethyl (4-methoxyphenyl)difluoromethylphosphonate (**3d**)

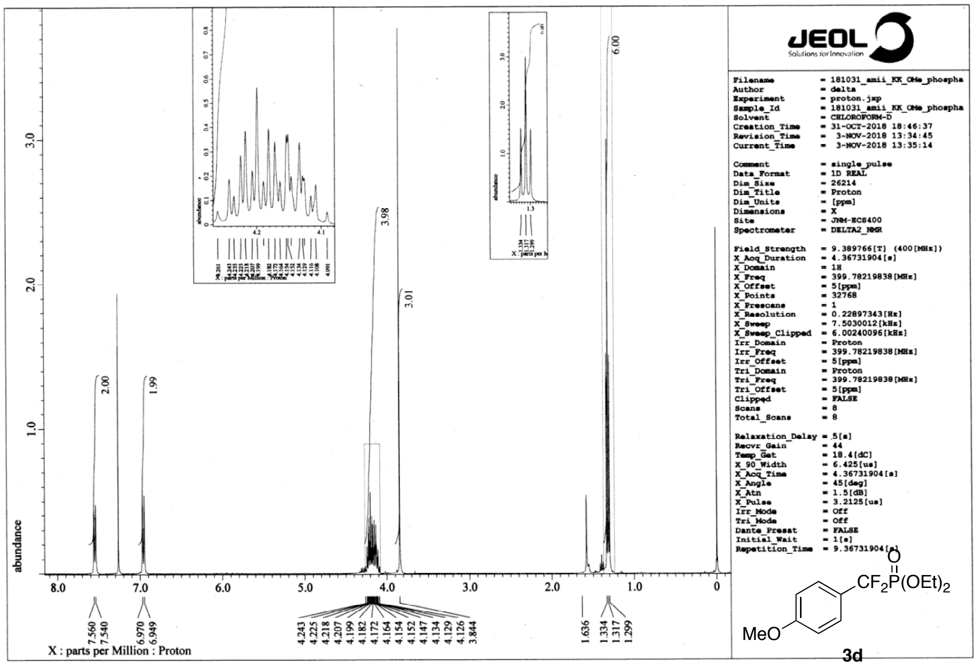

^{19}F NMR Spectrum of diethyl (4-methoxyphenyl)difluoromethylphosphonate (**3d**)

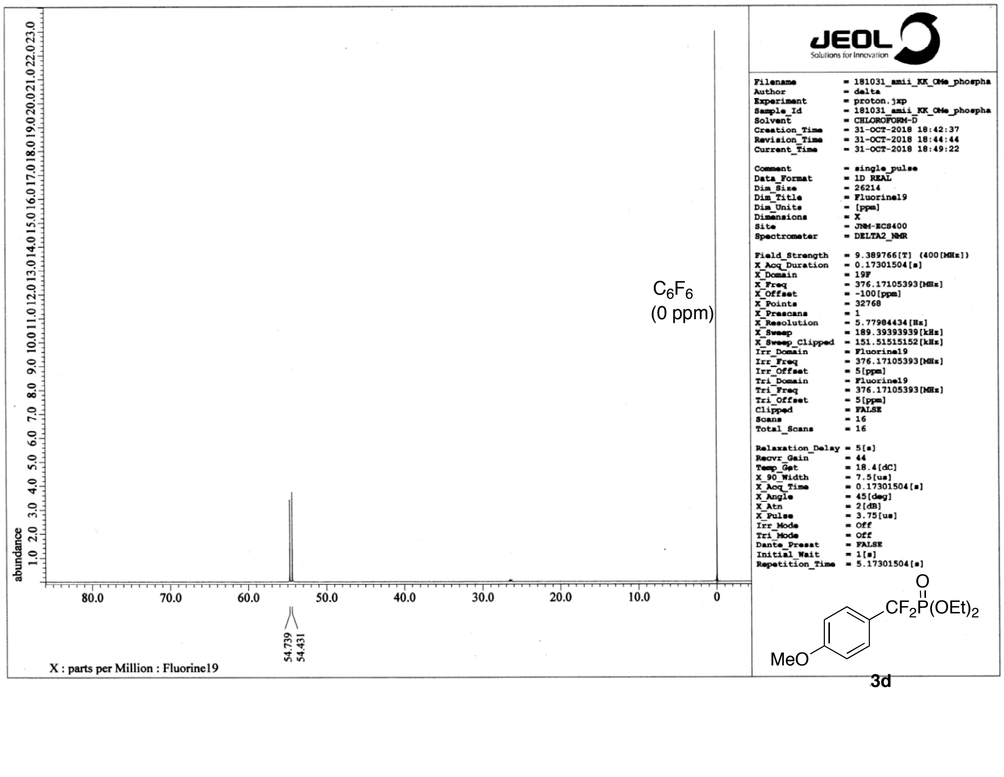

^{1}H NMR Spectrum of diethyl difluoro(naphthalen-1-yl)methylphosphonate (**3e**)

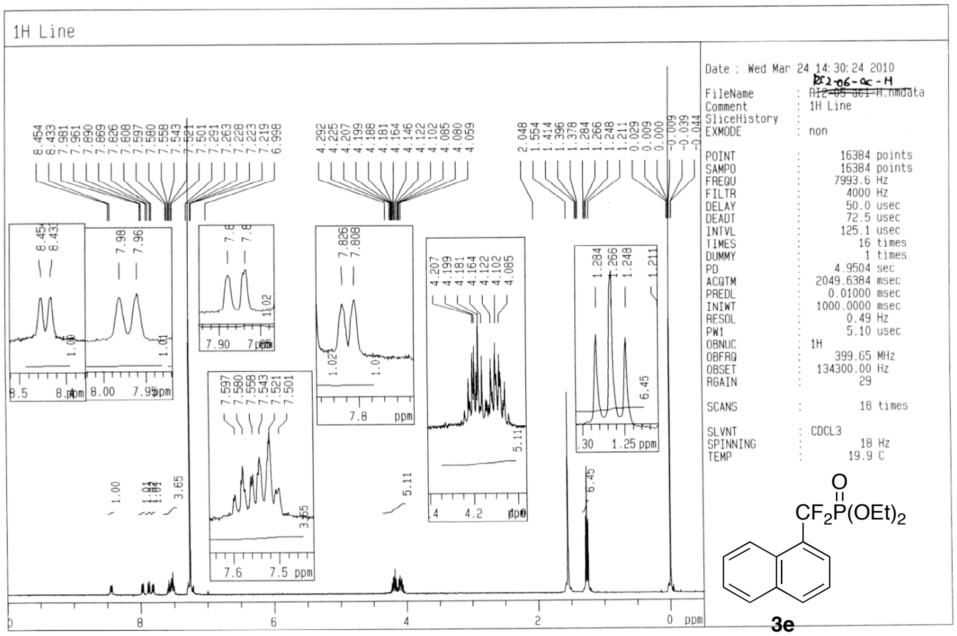

3e

^{19}F NMR Spectrum of diethyl difluoro(naphthalen-1-yl)methylphosphonate (**3e**)

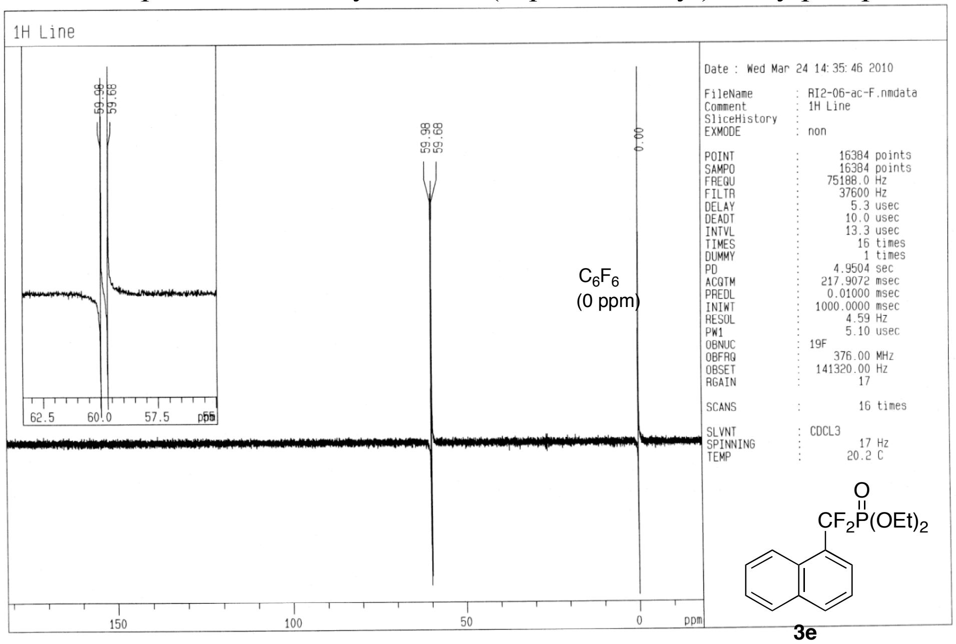

3e

^{1}H NMR Spectrum of diethyl difluoro(pyridin-2-yl)methylphosphonate (**3f**)

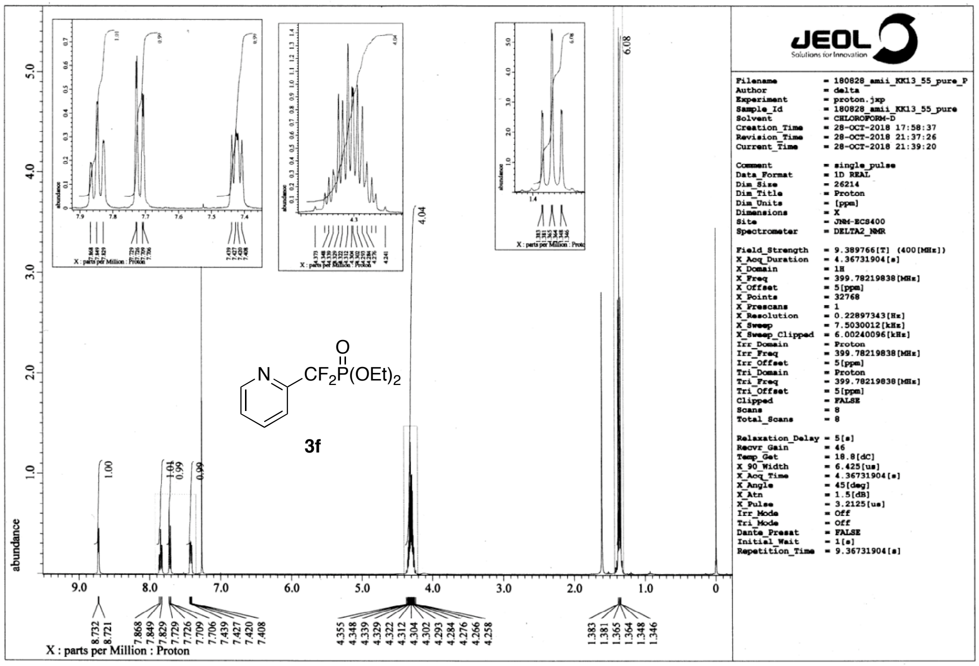

^{19}F NMR Spectrum of diethyl difluoro(pyridin-2-yl)methylphosphonate (**3f**)

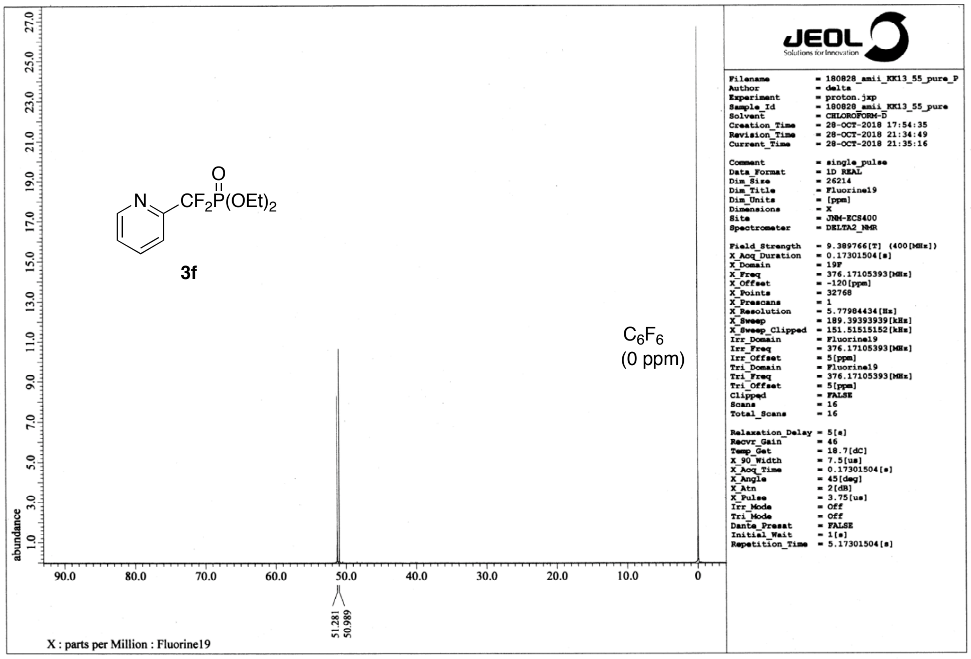

^{1}H NMR Spectrum of diethyl difluoro(quinolin-2-yl)methylphosphonate (**3g**)

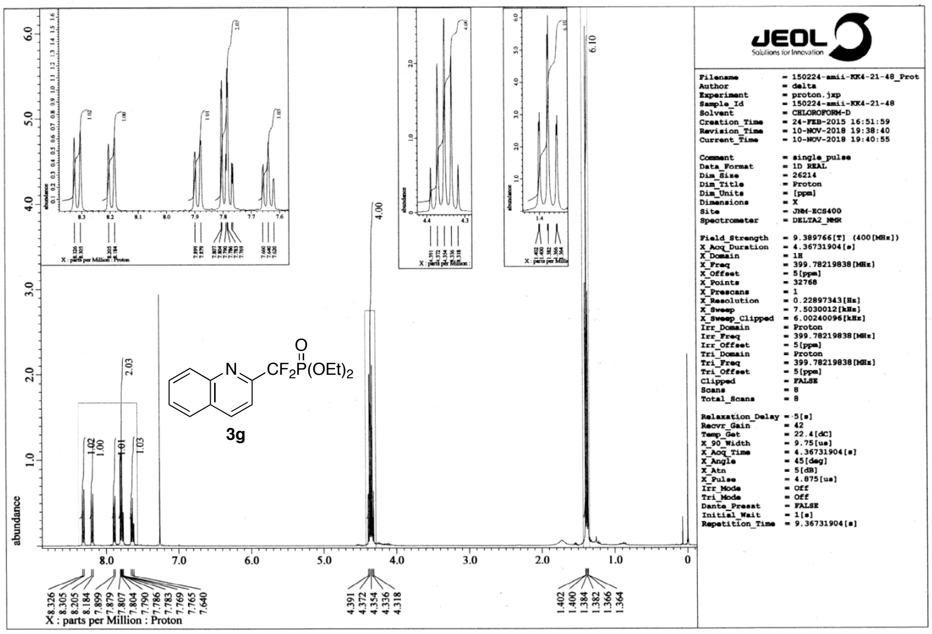

^{19}F NMR Spectrum of diethyl difluoro(quinolin-2-yl)methylphosphonate (**3g**)

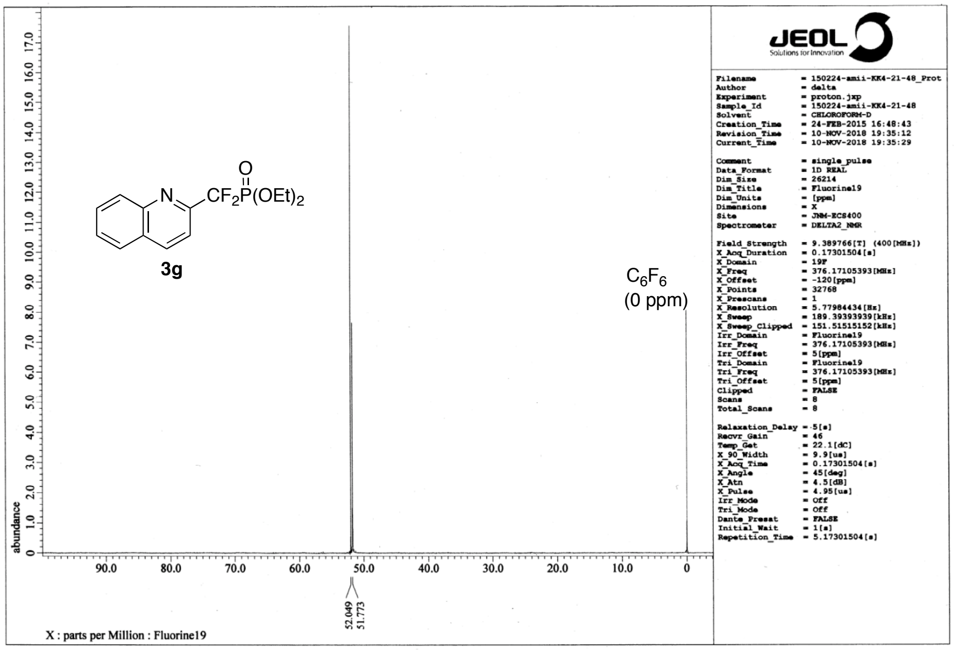

^{1}H NMR Spectrum of diethyl (3,4-dichlorophenyl)difluoromethylphosphonate (**3h**)

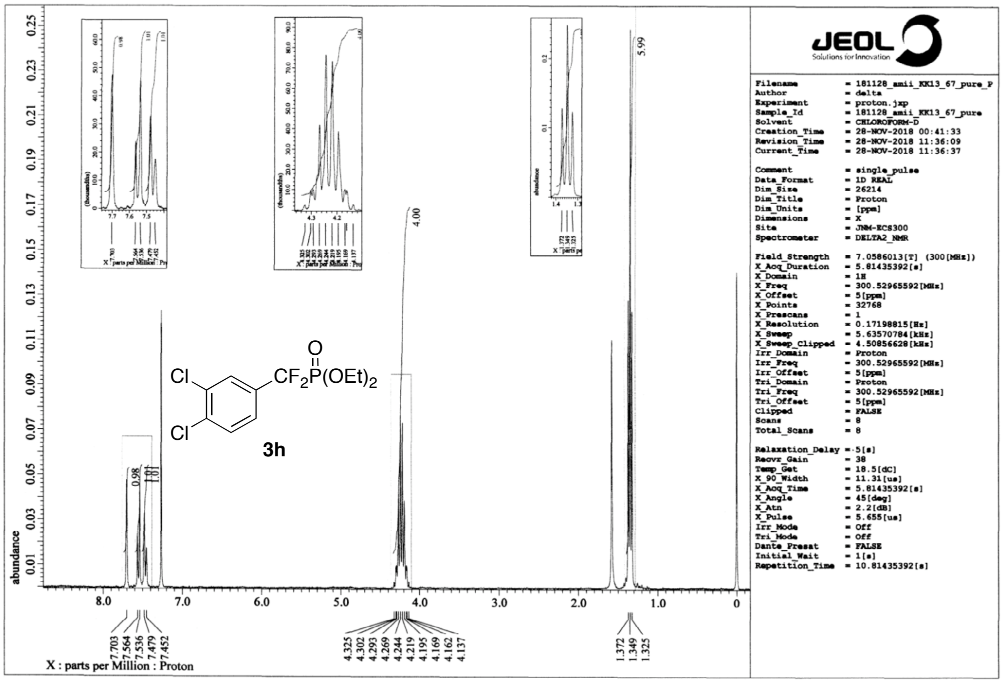

^{19}F NMR Spectrum of diethyl (3,4-dichlorophenyl)difluoromethylphosphonate (**3h**)

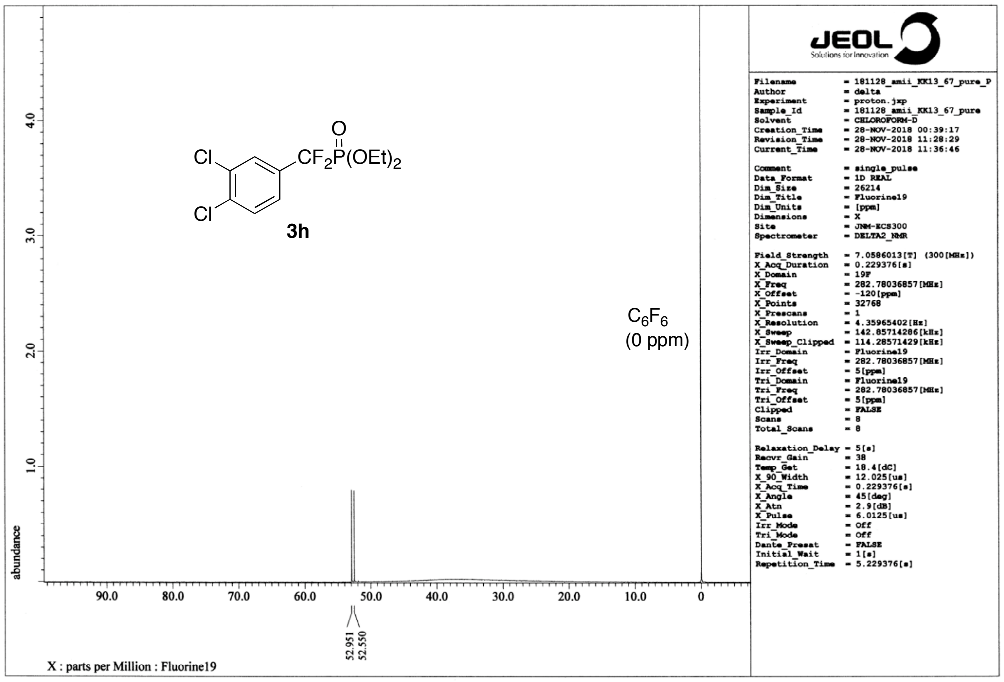

^{1}H NMR Spectrum of diethyl (4-bromophenyl)difluoromethylphosphonate (**3i**)

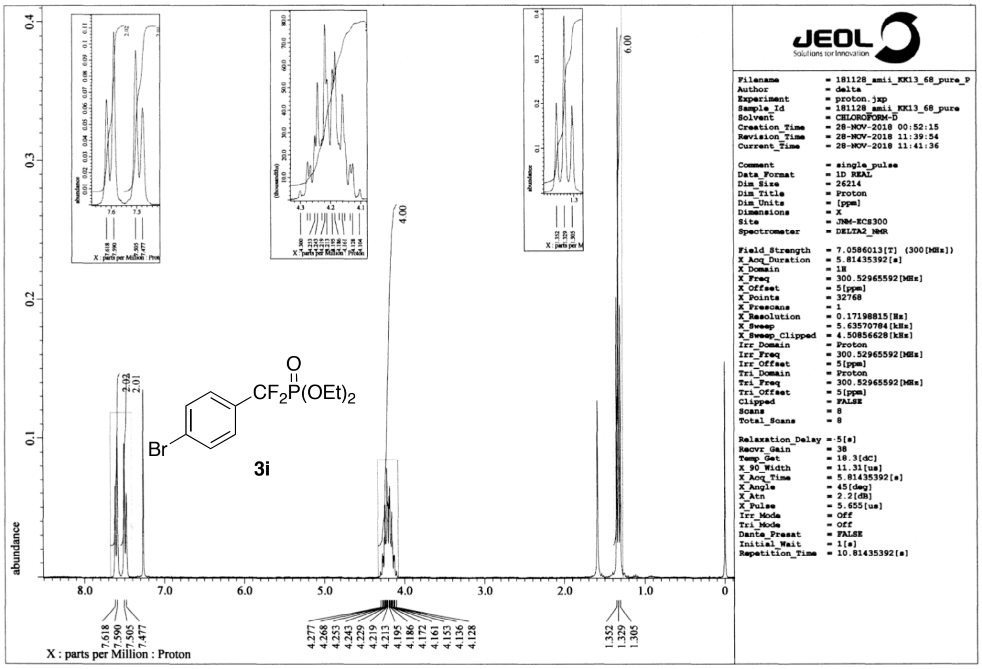

^{19}F NMR Spectrum of diethyl (4-bromophenyl)difluoromethylphosphonate (**3i**)

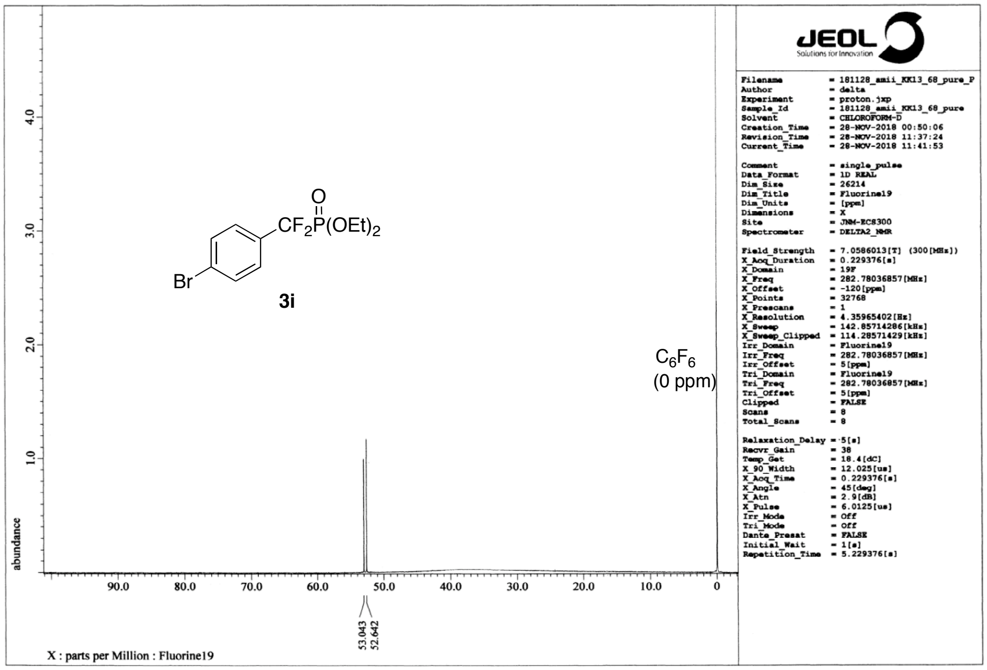

Supplement: Supplementary File 1 [file molecules-23-03292-s001.pdf]
